# Giant Intrauterine Cystic Malformation of Thumb

**DOI:** 10.21699/jns.v6i2.466

**Published:** 2017-04-15

**Authors:** Arushi Agarwal, Kamal Nain Rattan, Ankur Dhiman, Ananta Rattan

**Affiliations:** PGIMS Rohatak, Haryana, India

A newborn male child, born at 38-week gestation presented with complaint of a giant solitary cystic malformation of right thumb. Mother was non-smoker and non-alcoholic, with no history of any harmful drug or radiation exposure during pregnancy. She was primigravida and antenatal period was uneventful. On examination, a shiny, cystic swelling of 2.5cm x1.5cm x1.5cm was present instead of right thumb which had a nail at one end and the swelling was attached by a skin pedicle with hand (Fig.1A). No other gross congenital malformation was seen. Under regional anaesthesia, the cyst was excised. Histopathological examination revealed straw coloured fluid on cutting the cyst. Microsections showed a cyst lined with stratified squamous epithelium. Microsections examined from thumb showed fibrocartilaginous tissue revealing marked congestion, edema and hemorrhage along with an embedded cartilaginous tissue (Fig. 1B). 

Isolated congenital cystic lesion of thumb is a rare clinical entity. Radiological investigations usually do not lead to definitive diagnosis. Computed tomography and magnetic resonance imaging are helpful in delineating the anatomy of the soft tissue of the lesion, although seldom necessary to guide the treatment. [1] These lesions have multiple differential diagnosis epidermal cyst, enchondromatosis, lipoma, neurofibroma, fibrodysplasia, and constriction band. [2] In our case, an amniotic band might cause slow in-utero constriction and swelling of the thumb. No treatment is needed if cystic lesion is asymptomatic and does not interfere with hand functions but the surgical treatment is needed for symptomatic cysts causing interference with hand function. If cystic lesion involve thumb instead of other phalanx of hand then surgical intervention should be done very carefully. Always try to preserve thumb as much as possible due to its importance in hand functions. In our scenario, thumb was non-functional and cystic lesion was attached with tag of skin. Hence complete excision/amputation of cystic part of thumb was the only option of surgical treatment. 

## Footnotes

**Source of Support:** None

**Conflict of Interest:** None

## Figures and Tables

**Figure 1:**
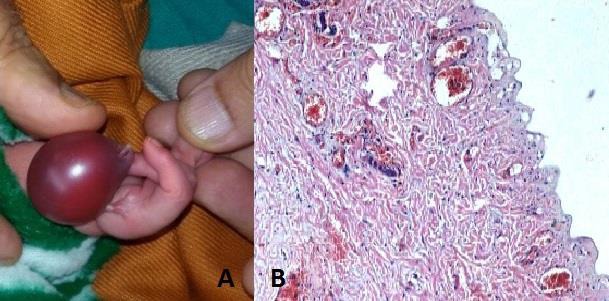
Thumb swelling. B) Histopathology.
